# Dreams and Nightmares in Patients With Obstructive Sleep Apnea: A Review

**DOI:** 10.3389/fneur.2019.01127

**Published:** 2019-10-22

**Authors:** Ahmed S. BaHammam, Aljohara S. Almeneessier

**Affiliations:** ^1^Department of Medicine, The University Sleep Disorders Center, College of Medicine, King Saud University, Riyadh, Saudi Arabia; ^2^The Strategic Technologies Program of the National Plan for Sciences and Technology and Innovation in the Kingdom of Saudi Arabia, Riyadh, Saudi Arabia; ^3^Prince Naif Health Research Center, King Saud University Medical City, King Saud University, Riyadh, Saudi Arabia; ^4^Department of Family and Community Medicine, King Saud University, Riyadh, Saudi Arabia

**Keywords:** sleep-disordered breathing, CPAP, REM sleep, post-traumatic stress disorder, hypopnea, emotion

## Abstract

Obstructive sleep apnea (OSA) can present with or provoke various psychological symptoms. In this article, we critically review studies that have examined dreams, dream recall, and dream content in patients with OSA. Obstructive events induce recurrent sleep fragmentation and intermittent desaturations in patients with OSA, which may trigger different parasomnias, including nightmares. Contradictory results have been reported concerning dreams in patients with OSA; while some investigators have reported less dreams in OSA patients, others have described that patients with OSA have increased dreams with emotional content, mainly violent and hostile content. Although there are reports of respiratory-related dream content in patients with OSA, most studies that have assessed the dream content of patients with OSA revealed that respiratory-related dream content was unusual. A clear association between post-traumatic stress disorders, comorbid OSA, and nightmares has been reported in several studies. Furthermore, an improvement in nightmare frequency with continuous positive airway pressure (CPAP) treatment has been shown. An inverse relationship between the severity of OSA reflected by the apnea-hypopnea index and dream recall has been demonstrated in several studies. Future studies should differentiate between patients with non-stage specific OSA and patients with rapid eye movement (REM) predominant OSA.

## Introduction

Obstructive sleep apnea (OSA) is a common sleep-related breathing disorder (SRBD). Among the middle-aged population, the prevalence of OSA syndrome ranges from 3 to 18% in men and from 2 to 17% in women. The prevalence of OSA increases with age, and it is projected that 28–67% of older men and 20–54% of older women have OSA syndrome (1–3).

The characteristics feature of OSA is recurrent occurrences of upper airway obstruction during sleep, which ranges from partial upper airway obstruction (hypopnea) or complete upper airway obstruction (apnea). The two major pathophysiological consequences of OSA are sleep fragmentation and arterial oxygen desaturation (4).

OSA is associated with adverse health outcomes, including decreased quality of life, insulin resistance, and increased risk for cardiometabolic disorders and mortality (5). Excessive daytime sleepiness (EDS) is a major consequence of OSA that often constitutes a risk factor for motor vehicle crashes (6). OSA can present with or provoke various psychological symptoms (7). Sleep fragmentation secondary to recurrent obstructive events and recurrent desaturations in patients with OSA may trigger parasomnias (8, 9).

In the nineteenth century, researchers hypothesized that recurrent oxygen desaturation during sleep may prompt the appearance of nightmares (10). In an older study, Boerner reported that blocking the nose and mouth with a cloth induced nightmares (11). Nevertheless, conflicting results have been reported regarding dreams in patients with OSA. Some studies have reported less dreams in OSA patients, and others have described that patients with OSA have increased dreams with emotional content, mainly violent and hostile. In this article, we critically review studies that have examined dreams, dream recall, and dream contents in patients with OSA.

## OSA and Emotional Regulation

Human behavior and emotions are regulated by the amygdala, locus coeruleus, and prefrontal cortex (12). Sleep, particularly rapid eye movement (REM) sleep, maintains brain homeostasis, which supports optimal social and emotional functioning (13). Current evidence supports the concept that sleep is critical for the formation of emotionally-valenced information throughout all stages of emotional regulation (12). Moreover, sleep is vital for the regulation of emotions. Hence, sleep disturbances in patients with OSA, such as slow wave sleep (SWS) and REM sleep disturbances, may pre-dispose patients to neurological and psychiatric disorders (12).

Though some researchers have reported less dreams in patients with OSA, others have described that patients with OSA have increased dreams with emotional content, mainly violent and hostile (14–17). It has been postulated that obstructive events during REM sleep may activate the limbic system, which may increase dreams with high emotional contents (15).

Moreover, OSA and psychiatric disorders have a bidirectional relationship, where the presence of one disorder could worsen the other. OSA is prevalent in psychiatric patients (7), especially those with depression (18, 19), anxiety (20), and post-traumatic stress disorder (PTSD). On the other hand, patients with OSA have a higher risk of developing psychiatric symptoms and comorbidities, in comparison to the general population (21). Moreover, patients with OSA have a higher rate of mood disorders, anxiety disorders, PTSD, and psychotic disorders, compared with non-OSA patients (18, 22). The coexistence of comorbid psychiatric disorder may interfere with dream content or nightmares (23, 24).

## Dream Recall in Patients With OSA

Studies have reported contradictory results with regards to dream recall in patients with OSA (10, 15, 25). The majority of previous studies that have assessed the relationship between OSA and dream recall has been retrospective. Whereas, some investigators reported less dream recall in patients with OSA and improvement of dream recall after treatment with continuous positive airway pressure (CPAP), others have demonstrated that OSA patients have increased dream recall frequency and increased dreams with emotional content, particularly aggressive and violent content (14–17). Table 1 presents an overview of studies that assessed dream recall in patients with sleep-disordered breathing

**Table 1:** A summary of studies that assessed dream recall in patients with sleep-disordered breathing.

**References**	**Study sample and protocol**	**Dreams assessment tools**	**OSA diagnosis measures**	**Conclusions**
Carrasco et al. (15)	20 consecutive patients with severe OSA and 17 healthy controls. PSG were recorded at baseline and during the CPAP titration night, 3 months after effective treatment and 2 years later in patients	Subjects were awakened 5–10 min after the beginning of the first and last REM sleep periods to measure percentage of dream recall, word count, thematic units, sleep architecture, and REM density	PSG	Dream recall was similar in patients at baseline and controls. Word count was higher in patients than in controls. Despite an increase in REM density, dream recall decreased the first months of CPAP and recovered 2 years later. Dream recall decreased with CPAP therapy and with normalization of sleep in OSA patients
Lovin et al. (26)	38 patients with OSA had a diagnostic study followed by a therapeutic study within 1–3 weeks	Dream recall was done immediately following spontaneous awakening from REM sleep	PSG	More patients recalled their dreams under CPAP than without CPAP
Schredl and Schmitt (27)	1,467 patients with OSA. And two representative samples from the general population	Questionnaire	PSG	Heightened dream recall frequency in patients compared to controls
Pagel and Kwiatkowski (28)	394 with severe OSA	A questionnaire with questions on dream and nightmare recall frequency	PSG	Both AHI and arousal index were significantly higher for the grouping reporting infrequent nightmare recall. Depressed nightmare recall may occur secondary to the REM sleep suppression that is known to occur in patients with significant OSA
Gorss and Lavie (25)	33 patients with OSA slept during two nights in the sleep laboratory. 16 were treated with CPAP) during the first night and 17 during the second. Patients were awakened for dream reports 10 min after the beginning of every REM period	Patients were asked upon awakening: “what was going through your mind before I woke you up?” Patients were also asked “how they felt in the dream” Dream reports were tape recorded.	PSG	After apneas, dream recall tended to be higher and dream reports were significantly longer
Schredl et al. (29)	762 patients who were diagnosed with different sleep disorders	Questionnaire	PSG	Dream recall frequency (DRF) of patients OSA did not differ from DRF in healthy controls. OSA parameters did not correlate substantially with DRF

Initial studies that assessed dream recall at home by retrospective questionnaire scales demonstrated lesser dream recall in patients with OSA than in healthy controls (14, 30). A subsequent study of 1,243 patients with OSA demonstrated higher dream recall in OSA patients than in a representative sample from the general population (31). A recent study of 1,467 patients with OSA who completed a dream questionnaire reported heightened dream recall frequency in patients compared to that in controls (27). However, several studies reported no correlation between OSA severity parameters [i.e., apnea hypopnea index (AHI) and desaturation index] and dream recall frequency (14, 17, 30, 32, 33).

Studies assessing the effects of CPAP therapy on dream recall have reported conflicting results. In a sample of patients (*n* = 33) already treated with CPAP for a minimum of 2 months, Gross and Lavie demonstrated that discontinuation of CPAP therapy for one night caused an increased AHI and enhanced dream recall, and dream reports were significantly longer on untreated nights (25). However, the investigators did not assess the recall of dreams before the initiation of CPAP treatment (25). Moreover, dream reports from the untreated nights and after apneas appeared to be more negative (25). However, it is possible that sleeping without CPAP therapy after 2 months of treatment may have caused these patients to worry, thereby enhancing the more negatively toned dreams which were not related to obstructive events.

One more study in patients with severe OSA reported that despite an increase in REM density after CPAP therapy, dream recall diminished after CPAP initiation both acutely and after 3 months (15). However, this finding was independent of REM density and the amount of REM sleep. The authors theorized that the decrease in sleep fragmentation following CPAP therapy could explain this finding (15). Moreover, the recovery increment of SWS proportion induced by CPAP has also been suggested as an explanation for the reduction in dream recall (15).

Gender differences in dream recall in patients with OSA have been reported recently by Schredl and Schmitt (27) with more recall observed in women, which is consistent with the findings of previous studies that reported gender differences in dream recall (34).

Carrasco et al. proposed two theories that may affect dream recall in OSA patients (15). The first theory is the cognitive impairment theory, where impairment of cognitive function in OSA patients may impair dream recall (35). The second contradictory theory is the frequent arousals theory, where frequent arousals and light interrupted sleep may increase dream recall (15).

Possible explanations for the decreased recall of dreams in some patients with OSA include the possibility that time spent awake after respiratory event-induced arousals is too short to allow dream recall (27). Another possibility is that cognitive deficits in patients with OSA may offset the proposed effects of frequent arousals on dream recall (27, 35). Future studies should account for the duration of arousals and obstructive events, cognitive function, and AHI during REM sleep and their impact on dream recall.

## Dream Contents

Exposure to stimuli during REM sleep can modify dream contents (36). The effects of external auditory, sensory, and olfactory stimuli during REM sleep have been shown previously (37–39). However, it remains unknown if internal pathological changes (e.g., obstructive apneas and hypopneas) affect dream content (27). Table 2 presents a summary of studies that assessed dream contents and nightmares in patients with sleep-disordered breathing.

**Table 2:** A summary of studies that assessed dream contents and nightmares in patients with sleep-disordered breathing.

**References**	**Study sample and protocol**	**Dreams assessment tools**	**OSA/Snoring diagnosis measures**	**Conclusions**
Gorss and Lavie (25)	33 patients with OSA slept during two nights in the sleep laboratory. 16 were treated with CPAP) during the first night and 17 during the second. Patients were awakened for dream reports 10 min after the beginning of every REM period.	Patients were asked upon awakening: “what was going through your mind before I woke you up?" Patients were also asked “how they felt in the dream” Dream reports were tape recorded.	PSG	Dreams after obstructive events were significantly more negative than dreams after healthy sleep. The results show no manifest stimulus incorporation in REM sleep dream reports
Di Pauli et al. (32)	63 patients with OSA (mild *n* = 50%, moderate, 21%, and severe 30%), and 13 subjects as a control group with primary snoring. Patients with OSA were older	Dream reports, and dream questionnaires were collected immediately after first morning awakening	PSG	No significant difference in respiratory-related dream topics between patients and controls The word count of dream reports did not differ between cases and controls. Breathing-related content was not increased in those with higher oxygen desaturation
Fisher et al. (17)	A prospective observational study of 47 patients with sleepiness and snoring. Participants were grouped into: sleepy snorers, AHI <5: *n* = 12; AHI 5–14.9, *n* = 14; AHI ≥ 15, *n* = 21	A morning diary concerning pleasantness/unpleasantness of their dreams for 10 days	A limited-channel home sleep study	All groups reported similar numbers of dreams and nightmares during the diary period. The AHI ≥ 15 group were significantly higher on dream unpleasantness than were the AHI <5 group. The variation in mean dream emotion decreased with increasing AHI
Schredl et al. (14)	444 patients with OSA and 941 healthy controls. Twelve patients had an additional diagnosis of a mental disorder	Nightmare frequency was measured by an eight-point rating scale administered before the first laboratory night	PSG	In general, the respiratory parameters as measures of sleep apnea syndrome severity did not correlate substantially with nightmare frequency Comorbid psychiatric disorders and an intake of psychotropic drugs were associated with heightened nightmare frequency
Carrasco et al. (15)	20 consecutive patients with severe OSA and 17 healthy controls. PSG were recorded at baseline and during the CPAP titration night, 3 months after effective treatment and 2 years later in patients	Subjects were awakened 5–10 min after the beginning of the first and last REM sleep periods and the investigators measured percentage of emotional content of the dream, word count, thematic units, sleep architecture, and REM density	PSG	Violent/highly anxious dreams were only seen in patients at baseline. Dreams in patients with severe OSA had an increased emotional tone and were longer. Violent/highly anxious dreams disappeared with treatment
Lovin et al. (26)	38 patients with OSA had a diagnostic study followed by a therapeutic study within 1–3 weeks	Dream recall was done immediately following spontaneous awakening from REM sleep, and was analyzed for dream content (word count, number of thematic units, and emotional content)	PSG	Dream content was less unpleasant under CPAP
Schredl and Schmitt (27)	1,467 patients with OSA. The control group consisted of 2,929 persons completing an online version of the questionnaire	The Mannheim Dream Questionnaire. For nightmares, the ICSD-3 definition was used. Nightmare distress was measured using a five-point scale	PSG	After controlling for age, gender, and dream recall frequency, patients reported less intense dreams, a less positive attitude toward dreams, and lower lucid dream frequency. Women tended to report more intense Dreams and more positive emotional tone
BaHammam et al. (40)	A prospective case-control study of 99 patients with OSA with nightmares and 124 patients with OSA without nightmares. Excluded patients with psychiatric disorders and those on medications that can cause nightmares.	Nightmares were diagnosed according ICSD 2005	PSG	REM-AHI and interrupted sleep at night were independent predictors of nightmares in the OSA patients. Nightmares disappeared in 91% of the patients who used CPAP compared with 36% of patients who refused to use CPAP
Pagel and Kwiatkowski (28)	394 with severe OSA	A questionnaire with questions on dream and nightmare recall frequency	PSG	Patients with higher AHI report a lower nightmare frequency. However, the study did not report the details of respiratory events during REM sleep in OSA patients with nightmares
Hicks and Bautista (41)	199 university undergraduates completed a questionnaire that assessed their level of snoring and the frequency of nightmares.	Questionnaire	Questionnaire	No significant relationship between snoring and disturbing dreams
Schredl (42)	The study assessed the correlations between snoring, and breathing cessation during sleep, and nightmare frequency in a sample of 444 healthy adults	Questionnaire	Questionnaire	Breathing pauses, but not snoring were associated with heightened nightmare frequency

An earlier study assessed 33 patients with OSA who slept during two nights in the sleep laboratory before and after CPAP therapy (25). Ten minutes after the beginning of every REM period, patients were awakened for dream reports. After awakening from REM sleep secondary to obstructive events, there was a trend toward increased dream recall (60 vs. 72%, *p* = 0.09) and dream reports were more elaborative (16 vs. 24 words, *p* = 0.05) than those after awakening from REM sleep without obstructive events (25). However, the apnea stimulus was not incorporated into the dream reports. Nevertheless, after apneas, dreams were significantly more negative than dreams after healthy sleep (*p* < 0.01) and included significantly more characters, activities, and social interactions (25). The authors proposed that the stress caused by apneas exerted only a global emotional influence on manifest dreaming (25).

Most studies assessing dream contents of patients with OSA revealed that respiratory-related dream contents were unusual (25, 31, 32, 43, 44). Nevertheless, in a prospective case-control study of 99 patients with OSA and nightmares (excluding patients with psychiatric disorders), BaHammam et al. reported that dream contents in most studied patients were related to suffocation (40). Examples of patients' reports included the following: “I dreamt that I fell in a deep well and was gasping for breath” (male, 40 years, AHI 45/h), “that a person was holding my neck preventing me from breathing” (female, 43 years, AHI 49/h), and “I dreamt of drowning in dark water and was gasping for breath” (female, 39 years, AHI 47/h) (40). Similarly, other authors reported respiratory-related dreams including “I am buried under the sand and fighting my way to a surface that I can't seem to reach. I wake gasping for air” (45); “I was lying in a coffin-dead-and cried: My god, please, don't let me die, I am still young and I have small children” (44); “I was diving without oxygen tanks and was gasping for breath. The way to the surface was far and I managed it just in time. After waking up I really gasped for breath” (14).

Differences between studies could be attributed to the dissimilarities in characteristics of the included group in each study (e.g., cognitive function, duration of REM sleep, AHI during REM sleep, and patients' awareness of the severity of their illness) and the adopted exclusion criteria (40), because patients' awareness of an illness and its complications may influence the emotional; contents of their dreams (27).

## OSA and Nightmares

The first description we could find suggesting a link between OSA and nightmares was written by Avicenna (980–1,037 C.E.) (46). He stated that people who sleep on the back have an open mouth as the muscles of the mouth and throat get too weak to maintain the normal position (suggesting sleep-disordered breathing), and further, he described that sleeping on the back can lead to stroke, paralysis, and nightmares (46, 47). Subsequently, investigators in the nineteenth century postulated that oxygen desaturation during sleep might cause nightmares (10, 11).

The International Classification of Sleep Disorders-third edition (ICSD-3) defined nightmares as “dreams with strong negative emotions that result in awakening from the dreams. The dream plot can be recalled very vividly upon awakening.” (48). The ICSD-3 has diagnostic criteria for nightmare disorder (nightmares), which includes three conditions that need to be met to fulfill the definition of nightmares (48). These criteria include: (1) repeated incidences of long, extremely dysphoric, and well-remembered dreams that usually involve threats to survival, security, or physical integrity; (2) the individual quickly becomes oriented and alert upon awakening from the dysphoric dreams; (3) the dreams and the resulting sleep disturbances result in clinically significant distress or impairment in social, occupational, or other important areas of functioning (48).

Previous studies have reported significantly lower frequency of nightmares in patients with more severe OSA reflected by a higher AHI (14, 28, 49). However, the majority of previous studies had a relatively small sample size, which affects statistical power and ability to account for confounding factors that may impact both OSA and nightmares.

A recent registry-based cross-sectional study of ~2,800 patients with OSA revealed that the prevalence of nightmares was 40.6% (49); however, the prevalence of nightmares in the controls [individuals without OSA]) was 50.1%, which was much higher than that reported in the general population (19.4%) (50). This reflects the fact that the study did not use a standard definition of nightmares, which may partly explain the discrepancies in the reported prevalence of nightmares in OSA patients. However, the study revealed that the prevalence of nightmares and sleep-related violence were inversely related to OSA severity (49).

“Nightmares” is one of the REM-related parasomnias, which occur during REM sleep. However, REM sleep is usually reduced in patients with severe OSA (40, 51). Therefore, Pagel and Kwiatkowski suggested that the reduction in nightmares in patients with severe OSA and an increased AHI may be related to the suppression of REM sleep in group of patients (28).

Nightmares have been reported in patients with REM-predominant OSA regardless of the degree of oxygen desaturation (40). In a prospective case-control study of 99 patients with OSA with nightmares (defined in accordance with the ICSD), BaHammam et al. demonstrated that although there were no significant differences in REM sleep duration or duration of apneas and hypopneas during REM and NREM sleep between OSA patients with and without nightmares, patients with nightmares had a significantly higher AHI during REM than patients without nightmares (40). However, no relationship was demonstrated between OSA severity reflected by AHI and SPO_2_ < 90% and the presence of nightmares (40). Logistic regression analysis identified the apnea-hypopnea index during REM sleep and interrupted nocturnal sleep as the independent predictors of nightmares in patients with OSA (40). This suggests that nightmares are possibly associated with the presence of apneas and hypopneas during REM sleep (40). It is possible that the patients who had frequent awakenings due to recurrent apneas/hypopneas during REM sleep recalled more nightmares. Interestingly, one study showed that the patients who are more difficult to arouse report lower nightmare frequencies (52). Nightmares in BaHammam et al. study were mostly related to suffocation (40).

Interestingly, in patients with good adherence to CPAP therapy, nightmares disappeared in 91%, compared with 36% of patients who did not adhere to CPAP (*p* < 0.001) (40). The authors postulated that recurrent obstruction during REM sleep rather than the duration of REM sleep may increase the vulnerability of patients with OSA to developing nightmares (40). This hypothesis is supported by previous studies, which demonstrated that one night of CPAP therapy caused a reduction in dreams and dream recall, though the amount and duration of REM sleep had increased (15, 25). Moreover, air hunger during wakefulness induced by breathing air that has high concentration of CO_2_ resulted in stimulation of the limbic/paralimbic brain regions (brain regions activated during REM sleep) (53). This may partly explain the increased nightmares in patients with higher obstructive events during REM sleep. Moreover, Carrasco et al. hypothesized that respiratory events during REM sleep cause stimulation of the limbic system; this, in turn, may generate dreams with high emotional contents (15).

The disturbances caused by sleep fragmentation in individuals with OSA can manifest on the patient emotionally by producing psychical symptoms, particularly, stress (54, 55). CPAP therapy has been shown to reduce stress in patients with OSA (55, 56). Therefore, it is possible that stress associated with OSA can be responsible for nightmares, and the reduction in stress with CPAP therapy results in a reduction in nightmares. This hypothesis needs to be tested.

## OSA and Post-traumatic Stress Disorder

According to 5th edition of the Diagnostic and Statistical Manual of Mental Disorders (DSM-V), post-traumatic stress disorder (PTSD) is explained by “*the development of a specific cluster of symptoms after exposure to a traumatic event that elicits a response of fear, helplessness, or horror*” (57).

Numerous studies have identified a wide range of comorbid sleep disorders in individuals with PTSD such as insomnia, nightmares, OSA, and sleep arousal, and enactment of dreams (58–60). A recent meta-analysis that aimed to identify the pooled prevalence of OSA in patients with PTSD on different AHI criteria showed an OSA prevalence of 76% (95% confidence interval [CI] = 44–93%) (AHI ≥ 5/h) and 44% (95% CI = 21–70%) (AHI ≥ 10/h), respectively (61).

Krakow et al. proposed that PTSD induces sleep fragmentation, which in turn disturbs the human upper airway and increases vulnerability to the consequent development of upper airway collapse (62). This may lead to the development of OSA, which may in turn worsen PTSD symptoms (62, 63). Moreover, it has been shown in some studies that sleep disturbances before traumatic events predict PTSD (64, 65).

A recent study of 59 patients with PTSD and 118 non-PTSD controls reported that weekly nightmare frequency was higher in patients with PTSD compared with the non-PTSD group before starting CPAP (66).

Previous studies have demonstrated a strong association between PTSD, comorbid OSA and nightmares, and an improvement in nightmare frequency with CPAP treatment (67). Germain et al. proposed that REM sleep enhances activation of the amygdala and reduces medial frontal cortex activation in patients with PTSD, which may worsen nightmares (68). As PTSD is associated with disturbances in both sleep and extinction memory, improving sleep quality may improve PTSD symptoms via strengthening naturally acquired extinction. It has been demonstrated that the hippocampus is susceptible to hypoxia, and decline in hippocampal activity correlates with the severity of PTSD (69). Nocturnal hypoxia in untreated OSA has been proposed as a possible trigger for PTSD symptoms including nightmares (66).

Previous studies have reported significant improvements in the frequency of nightmares and the symptoms of PTSD following CPAP therapy (70, 71). However, most of these studies had several limitations, including a retrospective design and small sample size. A recent prospective cohort study recruited 47 combat veterans with PTSD and OSA diagnosed by type one sleep study and reported that adherence to CPAP was associated with a reduction in nightmare distress and frequency (72). Treatment with CPAP resulted in a reduction of nightmare distress at 3 months follow-up in patients with both mild-to-moderate PTSD (*p* = 0.006) and severe-to-very-severe PTSD (*p* = 0.02) (72).

However, adherence to CPAP therapy in patients with PTSD and comorbid OSA remains a major problem. A recent meta-analysis revealed that the pooled estimate of regular use was lower in patients with PTSD and OSA than in those with OSA alone (61). The results of the pooled estimate of regular use were inferior in patients with PTSD and comorbid OSA compared with patients with OSA alone, with no significant heterogeneity (*I*^2^ = 0%), which points to the unfavorable influence of PTSD symptoms on CPAP compliance in patients with OSA (61).

Newer approaches that use holistic strategies to improve CPAP adherence in PTSD patients with comorbid OSA are needed. Approaches to enhance adherence include group education (73), cognitive-behavioral therapy (74), and motivation (75).

## Effects of Continuous Positive Airway Pressure (CPAP) on Dreams

There is a lack of consensus on the effects of CPAP therapy on dream recall and contents in patients with OSA. In a study of 20 patients with OSA (mean AHI of 73/h), Carrasco et al. reported a reduction in dream recall after one night of CPAP therapy accompanied by an increase in dream length and positively-toned dreams (15). Positively toned dreams, especially after long-term CPAP therapy, may be explained by the continuity hypothesis (whereby dreams express waking concerns and emotional preoccupations) because CPAP treatment is often seen as very beneficial in patients with OSA (76). However, another study of 38 patients OSA who underwent a diagnostic study followed by a second night on CPAP therapy reported a rebound of REM sleep accompanied by a quantitative increase in dream recall and a change in dream content (26). CPAP therapy resulted in a change in the dream content with a reduction in unpleasant content (50% without CPAP vs. 37.5% under CPAP) (26). A third study demonstrated that OSA patients with nightmares experienced an increase in sleep interruptions and had more obstructive events in REM sleep than those without nightmares. Notably, CPAP therapy resulted in a significant reduction in nightmares (40).

## Conclusion

Although the number of studies investigating dreaming in patients with OSA is relatively small, the literature is intriguing and shows that altered sleep physiology due to recurrent obstructive events, frequent arousals, and repeated desaturations may influence dream recall and dream content in some patients. Nevertheless, studies were inconsistent and reported conflicting results. Future studies addressing dreams and nightmares in patients with OSA should differentiate between REM-predominant OSA and non-stage specific OSA. Additionally, functional brain imaging, particularly of the limbic system during REM sleep in patients with OSA will facilitate understanding of the pathophysiology of dreams in this group of patients.

Moreover, future studies should measure the cognitive function of patients with OSA and dreams before and after CPAP therapy to assess the relationship between cognitive function and dream recall and contents. Similarly, assessment of mental function and stress before and after CPAP therapy is necessary to examine the relationship between improvement in psychical manifestations and dreams.

## Author Contributions

AB and AA have contributed equally to review of the literature, analysis, and writing of the manuscript.

### Conflict of Interest

The authors declare that the research was conducted in the absence of any commercial or financial relationships that could be construed as a potential conflict of interest.
